# Strain-Level microbial signatures and inferred functional alterations in infants with Food Protein-Induced Allergic Proctocolitis

**DOI:** 10.21203/rs.3.rs-7112201/v1

**Published:** 2025-08-27

**Authors:** Chen Goldstein, Itamar Lavy, Timothy Sun, Dena Ennis, Wayne G. Shreffler, Qian Yuan, Yamini V. Virkud, Victoria M. Martin, Moran Yassour

**Affiliations:** The Hebrew University of Jerusalem; The Hebrew University of Jerusalem; The Hebrew University of Jerusalem; The Hebrew University of Jerusalem; The Hebrew University of Jerusalem; The Hebrew University of Jerusalem; The Hebrew University of Jerusalem; The Hebrew University of Jerusalem; The Hebrew University of Jerusalem

## Abstract

The complex relationship between the gut microbiome and immune system development during infancy is thought to be a key factor in the rising rates of pediatric allergic diseases. Food protein-induced allergic proctocolitis (AP), the earliest identified form of non-IgE-mediated food allergy in infants, occurs at the mucosal surface where dietary proteins, intestinal microbes, and immune cells directly interact, and increases the risk for life threatening IgE-mediated food allergy, making it an important model for understanding early food allergic disease development. The question of how specific microbial compositions and functional pathways contribute to AP development and progression remains poorly understood.

Here we show that infants with AP exhibit microbial compositions that differ from unaffected controls, characterized by enrichment of *Escherichia coli* and *Bifidobacterium bifidum* during early life, including pre-symptomatic stages, while protective species like Bifidobacterium breve and *Klebsiella* species are more abundant in unaffected controls. Strain-level analyses uncovered additional disease-linked patterns, particularly *Lacticaseibacillus rhamnosus* strains showed strong association with probiotic use and predominantly found in infants with AP. These findings reveal disease-associated microbial signatures that can sometimes be detectable before clinical symptoms emerge, and demonstrate that strain-level differences within *E. coli* populations may represent AP-specific lineages with distinct gene content profiles that were not previously recognized. Genes for biofilm formation and cell adhesion in *E. coli*, for example, were particularly enriched in AP-associated clades. Short chain fatty acid (SCFA) and other functional pathways were also associated with AP, including reduced SCFA production during the symptomatic phase, and then a potentially compensatory increased production following AP resolution.

Our results provide the first comprehensive strain-level characterization of the gut microbiome in AP, and functional implications, and establish a foundation for future efforts to identify early microbial biomarkers and potential interventional targets for AP. This work advances our understanding of how specific microbial taxa and functional pathways may contribute to non-IgE-mediated food allergies and opens new avenues for microbiome-targeted therapeutic approaches as well as novel prevention targets for IgE-mediated food allergies

## Introduction

The complex relationship between the gut microbiome and immune system development during infancy is thought to be a key factor in the rising rates of pediatric allergic diseases, with particular interest in food allergy[1–12]. Food protein-induced allergic proctocolitis (AP)[13–15]is often the earliest commonly identified form of non-IgE-mediated food allergy in infants, and contrary to earlier dogma is associated with twice the odds of developing IgE-mediated food allergies[16, 17]. Occurring at the mucosal surface where dietary proteins, intestinal microbes, and immune cells directly interact, AP is an important model for study of food allergy development. Understanding the microbial and immunological factors involved in AP could provide valuable insights into the mechanisms underlying these early allergic manifestations and uncover opportunities for intervention or prevention of food allergies.

Emerging evidence suggests that microbial fermentation and its metabolites, particularly short-chain fatty acids (SCFAs) such as butyrate, acetate, and propionate, play a role in immune development and tolerance[18–20]. Indeed, SCFAs are known to contribute to maintaining intestinal barrier integrity and may support regulatory T cell differentiation, potentially influencing allergic risk[18, 19]. Among the various fermentation pathways, acetyl-CoA to butyrate production and succinate fermentation stand out for their associations with gut homeostasis and immune modulation[18, 21, 22]. For example, butyrate production via the acetyl-CoA pathway is linked to the modulation of inflammation and epithelial integrity, while succinate, another fermentation intermediate, can negatively affect immune responses through its signaling properties[22, 23]. Little is known about the SCFA milieu in infants with AP, but disruptions in SCFA-producing pathways—including reduced butyrate synthesis and changes in succinate metabolism—have been suggested to play a role in the pathogenesis of AP and other allergic manifestations[24–29]. Understanding these microbial functional changes may therefore offer further insights into the interactions between the gut microbiome and the development of allergic diseases in infancy.

*Bifidobacterium* is one of the earliest colonizers of the human gut and is associated with a range of health benefits, and several *Bifidobacterium* strains are used as probiotics[30]. In particular, *Bifidobacterium longum* has been shown to have anti-inflammatory effects, largely attributed to its ability to increase levels of SCFAs in the gut among other mechanisms. These effects allow its potential use as an adjuvant in the treatment of allergic diseases[31]. Furthermore, studies have reported that the loss of *Bifidobacterium* species in the infant gut (particularly in developed countries) is associated with a higher incidence of allergic and autoimmune diseases[32–35]. Among *Bifidobacterium longum* subspecies, *Bifidobacterium longum subsp. infantis* (*BL. infantis*), which is a key utilizer of human milk oligosaccharides (HMOs), is commonly associated with a healthy infant gut microbiome[36]. Recent developments have enabled the quantification of the various subspecies of *B. longum* from metagenomic sequencing[37], however it is still unknown whether BL. infantis abundance is associated with AP in the infant gut.

We previously conducted a comprehensive longitudinal analysis of the microbiome in infants with AP, including samples collected prior to symptom onset using 16S rRNA ribosomal gene sequencing[38],Those analyses found several taxonomic differences in infants with AP, including significant enrichment of *Enterobacteriaceae* early in life in infants with AP[38]. To better understand these findings at a species and functional level, we have now conducted more comprehensive longitudinal analyses using metagenomics. Metagenomics[39] applies a suite of genomic technologies and bioinformatics tools to directly access the genetic content of entire communities of organisms. This new information enables us to conduct a more in-depth analysis at the species & strain levels, using higher-resolution sequencing data to gain deeper insights into the microbiome of infants at various stages of the allergy and the differences between infants with and without AP. In this study, we apply metagenomic techniques to conduct detailed taxonomic analysis (down to the strain-level), focusing on tracking and identifying microbial changes in infants over time and identifying features that particularly differ in infants developing AP compared to those who do not. By examining these variations, we aim to gain insights into potential microbial contributors or protective factors associated with AP development and progression along the allergic march in early childhood.

## Results

### Cohort Description

**Figure 1:**
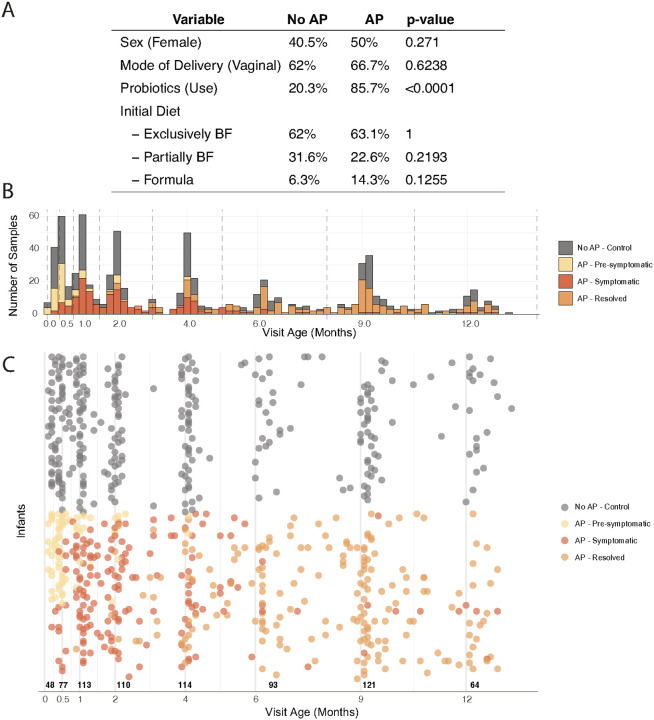
Study design, clinical characteristics, and longitudinal sampling. (**A**) Overview of key clinical and environmental features assessed in this case-control subset of the GMAP cohort, including infant sex, mode of delivery, initial feeding status (exclusive, partial, or formula feeding), and probiotic exposure during the first year of life. P-values for group comparisons were calculated using Fisher’s exact test (**B**) Number of samples analyzed across the first year, binned by the age of the infant at the time of sample collection, and colored by whether the infant was unaffected or had AP. Samples from infants with AP are colored by their symptom state at collection (pre-symptomatic, symptomatic, or resolved). (**C**) Sample map showing the analyzed longitudinal samples, plotted by infant age at collection and colored by symptom state. Each row represents one infant, and each dot represents one stool sample. Vertical dashed lines indicate the age bins used in downstream analyses. The x-axis labels represent the scheduled clinic visit ages; each age bin includes samples collected slightly before or after the planned visit, as described in the Methods.

To investigate the potential role of the infant gut microbiome in food protein-induced allergic proctocolitis (FPIAP, hereafter referred to as allergic proctocolitis or AP), we analyzed a total of 740 stool samples from 163 infants (84 infants with AP and 79 infants without AP) from the GMAP cohort[17, 38], each with robust longitudinal sampling (median 4 samples per infant; Fig. 1A; Methods). Across all samples, the median age at collection was 3.9 months (IQR: 1.1–8.0). For infants with AP, three clinically defined disease states were identified based on medical documentation: Pre-symptomatic (prior to onset), Symptomatic (during active disease), and Resolved (post-resolution). Samples within these phases were collected at median ages of 0.5 months (IQR: 0.3–0.9), 2.0 months (IQR: 1.1–4.1), and 8.6 months (IQR: 6.1–9.7), respectively (Fig. 1B–C).

All samples underwent deep, paired-end metagenomic sequencing (median 14 ~ million paired reads per sample), enabling species- and strain-level taxonomic profiling. Demographic and clinical characteristics were largely balanced between groups (Fig. 1A). Overall, 45% of infants were female, 65% were delivered vaginally, and their initial diet (at infant’s first outpatient visit) consisted of 63% exclusively breastfed, 27% partially breastfed, and 10% formula-fed. Group-level comparisons revealed no significant differences in sex distribution (50% vs. 40.5%, p = 0.271) or mode of delivery (66.7% vs. 62%, p = 0.6238). Initial diet (median age of 0.4 months) was also comparable across groups, with similar rates of exclusive breastfeeding (63.1% vs. 62%, p = 1.0), partial breastfeeding (22.6% vs. 31.6%, p = 0.2193), and formula feeding (14.3% vs. 6.3%, *p* = 0.1255). The only significant difference was a substantially higher rate of probiotic use in the first year among infants with AP (85.7% vs. 20.3%, p < 0.0001).

### Age-dependent maturation and stability of the infant gut microbiome

**Figure 2:**
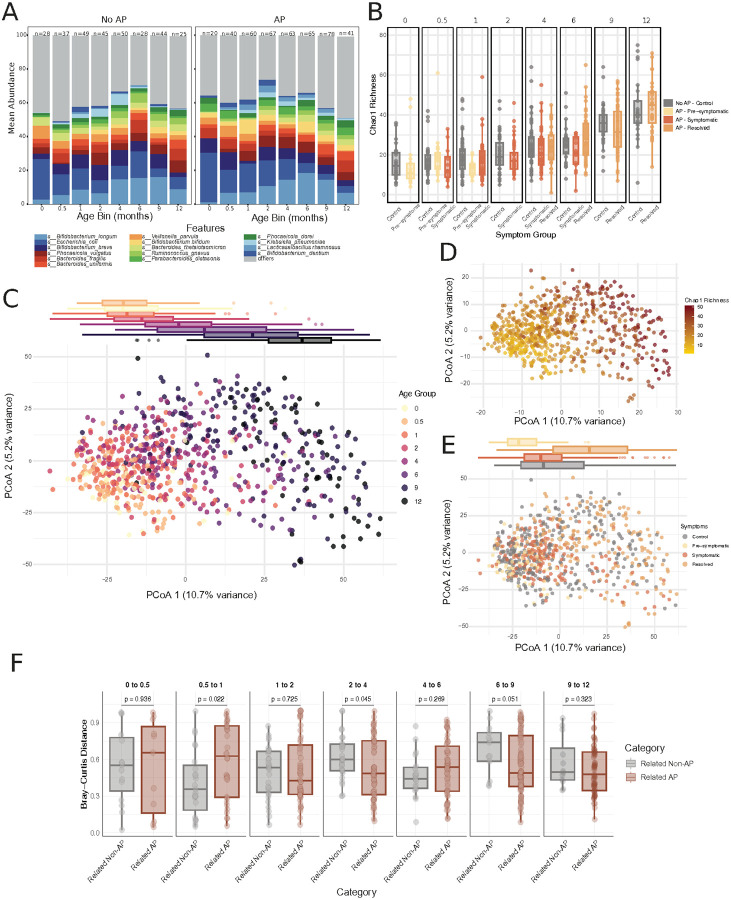
Overview of microbiome composition, structure, and taxonomic differences across the first year of life. **(A)** Composition plots displaying the mean relative abundance of the 15 most prevalent species-level taxa in infants with AP (right) and without AP (left), stratified by age. (**B**) Alpha diversity measured by Chao1 richness index in infants without AP and in infants with AP across clinical stages (pre-symptoms, symptomatic, resolved) and age bins. (**C–E**) Principal coordinates analysis (PCoA) based on Aitchison distance: (**C**) samples colored by visit age, highlighting age-related microbial structuring over time; (**D**) samples colored by Chao1 richness, illustrating increasing diversity throughout the first year; (**E)** samples colored by symptom group, with box plots summarizing distribution along the first principal coordinate. The first two axes explain 10.7% and 5.2% of the variance, respectively. (**F**) Within-infant sample dissimilarity in infants with AP versus infants without AP, calculated across successive age intervals.

We first examined the overall microbial composition across all infants during the first year of life. At both the genus and species levels, there were no major compositional differences between infants with AP and those without AP (Fig. 2A; **Supp. Fig, 1A**). *Bifidobacterium, Bacteroides, Escherichia*, and *Clostridium* were consistently among the most abundant genera, and dominant species included *Bifidobacterium longum, Escherichia coli, Bifidobacterium breve*, and *Bacteroides fragilis*, with broadly similar distributions between groups—mirroring known early-life colonization patterns.

Microbial richness increased significantly with age. Both Chao1 and Shannon diversity indices were positively correlated with age (Pearson r = 0.615 and 0.560; p = 3.76×10^−78^ and 2.27×10^−62^, respectively; **Supp.** Figure 1B-C), and no significant differences in richness were observed between symptom groups (Fig. 2B; **Supp.** Figure 1D). Principal coordinate analysis (PCoA) based on Aitchison distance further confirmed that age was the dominant source of variation in microbiome composition across the cohort (Fig. 2C–D; **Supp.** Figure 1E), while no clear separation was observed based on symptom group (Fig. 2E).

To assess microbial community stability, we compared longitudinal sample similarity within infants to that between infants. As expected, within-infant samples were consistently more similar than between-infant samples (all comparisons p < 10^−5^), and the variability among within-infant samples decreased over time, indicating greater compositional stability at later ages. (**Supp.** Figure 1F, **Supp**. **Table 1**).

We next asked whether microbiome stability differed by disease status. Comparing within-infant similarity between infants with and without AP across key age transitions revealed that infants with AP showed significantly less stable microbiomes between 2-week and 1-month samples (p = 0.022; Fig. 2F). In contrast, within-infant similarity was higher in infants with AP during later transitions (2–4 months and 6–9 months; p = 0.045 and 0.51, respectively), though these patterns were less consistent and occurred after symptom onset.

### Temporal Colonization Dynamics of Bifidobacterium longum Subspecies in Infancy

**Figure 3:**
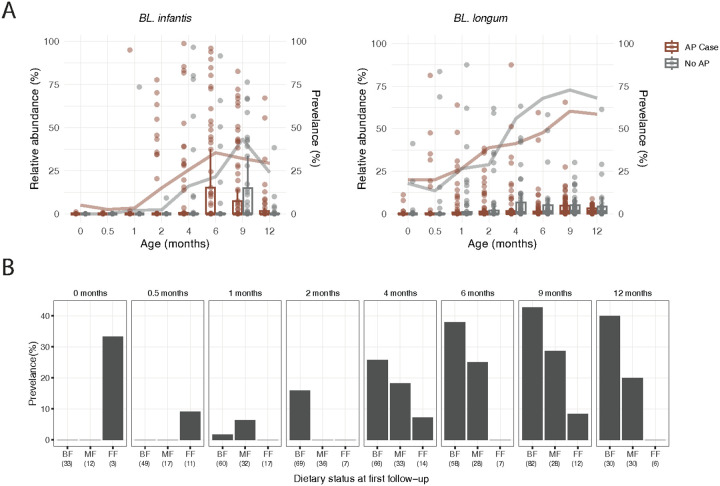
*B. longum* subspecies relative and prevalence over time. **(A)** Relative abundance of *BL. infantis* and (**B**) *BL. longum* over time, divided into AP and non-AP cases. The line represents prevalence over time. (**C**) Prevalence of *BL. infantis* at different time points in infants who were breastfed (BF), mixed fed (MF), or formula fed (FF). Numbers in parentheses indicate the number of infants in each category.

Given the important functional role of *Bifidobacterium* species in early-life nutrition and gut colonization, we next sought to investigate the temporal dynamics of the various *B. longum* subspecies colonization. We focused our analysis on the *Bifidobacterium* species that are associated with a healthy infant gut microbiome, searching for differences between infants with and without AP. Consistently with our previous findings in other cohorts[37], we found that *Bifidobacterium longum (BL.) subsp. infantis* was not detected at birth but began to emerge around two months of age, reaching a peak prevalence of 36% by nine months (Fig. 3A). This delayed pattern of colonization was observed in infants with and without AP (Fig. 3A). Although *BL. longum* also increased in prevalence over time, it started at a higher level at birth (18%) and reached 64% by nine months (Fig. 3B). As expected, feeding mode at birth had a significant effect on the likelihood of *BL. infantis* colonization (Fig. 3C). Beginning at two months, when *BL. infantis* colonization became more common, infants who had been exclusively breastfed at birth were more likely to harbor *BL. infantis* compared to those who were partially breastfed or formula-fed. Notably, partially breastfed infants also had a higher likelihood of colonization than formula-fed infants, suggesting a dose-dependent relationship between early breastfeeding and *BL. infantis* establishment (Fig. 3C).

### Taxonomic Differences Between Infants With AP and Infants Without AP

**Figure 4:**
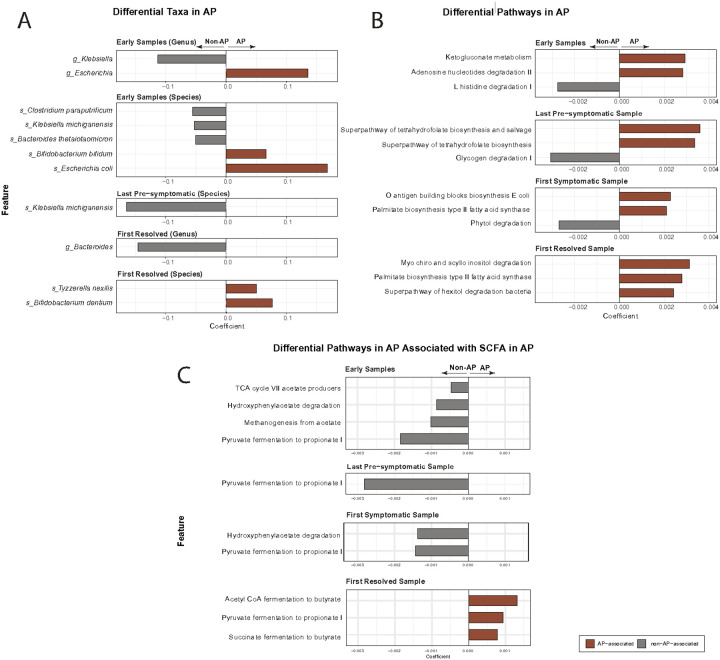
Differential abundance of microbial taxa and functional pathways associated with AP across infant subgroups. **(A)** Multivariable linear modeling of top genera and species identifies differentially abundant taxa between infants with allergic proctocolitis (AP) and unaffected controls across key clinical stages: early samples (0–2 months), last pre-symptomatic sample, first symptomatic sample, and first resolved sample. Positive coefficients indicate enrichment in AP; negative coefficients indicate enrichment in controls. (**B**) Linear model analysis of microbial metabolic pathways across the same clinical stages as in (A). The top three pathways with the largest absolute effect sizes are shown for each timepoint. (**C**)A focused view of fermentation-related pathways involved in short-chain fatty acid metabolism, including acetate, succinate, and butyrate production. “Acetyl-CoA fermentation to butyrate” refers specifically to the Acetyl-CoA → butyrate pathway II variant.

To identify compositional differences in the infant gut microbiome between infants with AP and infants without AP, we performed multivariable linear modeling at both the genus (**Supp. Table 2**) and species levels (**Supp. Table 3**), accounting for longitudinal samples, age, probiotic use, and feeding. In examining the earliest age window (0–2 months), we observed a clear enrichment of *Escherichia* in infants with AP (coefficient = 0.136; p = 0.016; q = 0.068), driven principally by *Escherichia coli* (coefficient = 0.168; p = 0.005; q = 0.035). Another species enriched in infants with AP was *Bifidobacterium bifidum* (coefficient = 0.067; p = 0.022; q = 0.092; Fig. 4A). In contrast, we found decreased abundance in infants with AP for *Klebsiella* species (coefficient = − 0.114; p = 0.003; q = 0.021) and *Clostridium paraputrificum* (coefficient = − 0.055; p = 0.013; q = 0.059; Fig. 4A).

In addition to predefined age ranges, we also examined microbial differences at time points related to disease progression. For example, when comparing the last sample prior to symptom onset (last pre-symptomatic sample; Methods), we found that *Klebsiella michiganensis* abundance was decreased in infants with AP (coefficient = − 0.165; p = 0.010; q = 0.247; Fig. 3A). Similarly, in the first samples following symptom resolution (first-resolved sample; Methods), we observed decreased abundance of *Bacteroides* species (coefficient = − 0.146; p = 0.040; q = 0.240) and increased abundance of *Bifidobacterium dentium* (coefficient = 0.077; p = 0.018; q = 0.212) in infants with AP (Fig. 4A).

We additionally examined species highlighted in prior literature as being associated with either IgE- or non-IgE-mediated cow’s milk allergy, including members of Clostridial clusters XIVa and IV (e.g., *Clostridium coccoides* [*Blautia coccoides*], *Eubacterium rectale, Roseburia inulinivorans, Faecalibacterium prausnitzii, Ruminococcus bromii*), as well as *Collinsella aerofaciens, Phascolarctobacterium faecium, Anaerostipes caccae*, and *Akkermansia muciniphila*. While many of these species were detected in our cohort, none showed statistically significant differences between infants with and without AP, in the early samples (0–2 months), nor across symptom phases (last pre-symptomatic, first symptomatic, or first resolved samples). These findings suggest that despite their known roles in fermentation, immune modulation, or associations with allergy in other contexts, these specific taxa do not appear to distinguish AP in our cohort. The full list of comparisons with statistics is found in **Supp. Table 3.**

### Microbial Functional Pathway Differences Associated with AP

To better understand potential microbial functional differences associated with allergic proctocolitis (AP), we examined metagenomic pathway abundances across symptom-defined and age-matched subgroups. Several fermentation-related and immune-relevant pathways showed differences between infants with and without AP (Fig. 4B–C; **Supp. Table 4**).

A significant difference between infants with AP and those without in the first symptomatic sample subset was found in the O-antigen building blocks biosynthesis pathway of *E. coli*. O-antigen is the outermost side chain of lipopolysaccharide (LPS), which is known to be variable and influence immune development and Th1/Th2 balance[20, 27] (coefficient = 0.0023, *p* = 0.00027, *q* = 0.008).

One pathway, pyruvate fermentation to propionate I, was consistently reduced in AP infants during early, pre-symptomatic, and symptomatic stages (coefficients − 0.0028 to − 0.0014, *p* = 0.003–0.023, *q* < 0.13), with a rebound following symptom resolution (coefficient = 0.00094, *p* = 0.077, *q* = 0.22), suggesting a dynamic association with disease progression.

Following symptom resolution, we also observed increased abundance of succinate fermentation to butyrate (coefficient = 0.00078, *p* = 0.0114, *q* = 0.061) and acetyl-CoA fermentation to butyrate II (coefficient = 0.00132, *p* = 0.0601, *q* = 0.188) in the AP group. These observations are consistent with patterns of altered short-chain fatty acid metabolism, and previous literature suggesting increasing butyrate is a biomarker of symptom resolution[25].

In contrast, phytol degradation and glycogen degradation I were less abundant in AP samples. Phytol degradation was reduced in the first-symptomatic (coefficient = − 0.00270, *p* = 1.27×10^−5^, *q* = 0.00083). Glycogen degradation I was lower in the last pre-symptomatic samples (coefficient = − 0.00146, *p* = 0.0064, *q* = 0.0206).

While these functional differences are observational, they point to variations in microbial metabolism between infants with and without AP, particularly in pathways related to fermentation and carbohydrate utilization. These differences may relate to disease pathogenesis (particularly those in the presymptomatic period) and provide opportunities for future interventional and prevention targets.

### Strain-Level Analysis highlights AP-associated microbial strains

We next explored strain-level differences to further identify potential variations between AP and unaffected infants. First, we generated phylogenetic trees from metagenomic data using species-specific marker genes. We focused our analysis on species that showed significant results in the species-level analysis or showed strong signals in the taxonomic differences analysis.

**Figure 5:**
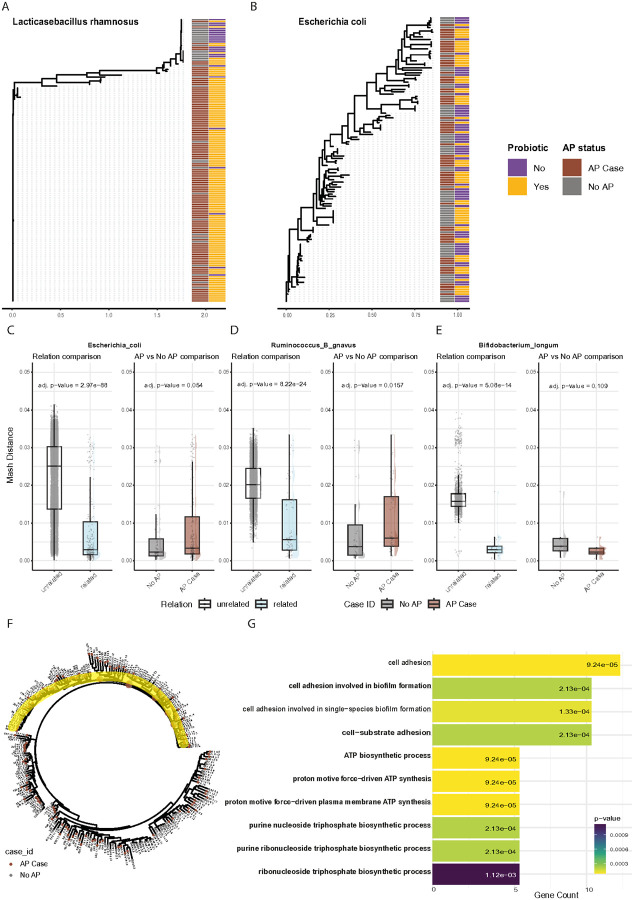
Strain level analysis and gene enrichment for AP-associated strains. (**A-B**)Marker-gene based phylogenetic trees displaying strains of *Lacticaseibacillus rhamnosus* (A) and *Escherichia coli* (B) in samples from 1 week to 1 year old. Colored by probiotic use in the first year (Methods) and by AP status. (**C-E**) MASH distances between MAGs of unrelated and related (from the same infant) samples (left), and within the related samples, separated to infants with AP (dark red) and infants without (AP; right). MAGs were compared within species for *E. coli* (C), *Ruminococcus B gnavus* (D), and *Bifidobacterium longum* (E). (**F**) A phylogenetic tree of *E. coli* MAGs, using functional profiling from Roary[55], colored by AP status (colors as in C-E). Pie-charts depict the proportion of infants with and without AP in the subtree of the given internal node, and are shown only when the disease status of the subtree significantly differs from the even distribution (50/50) with P-value < 0.05. Yellow highlights the examined MAGs, which are enriched for infants with AP. (**G**)Functional enrichment of biological processes, as defined in the EcoCyc database[59], showing top 10 significant results.

Due to the high prevalence of probiotic use in infants with AP, one of the striking strain-level differences was found in the *Lacticaseibacillus rhamnosus* species. We conducted a strain-level analysis of *L. rhamnosus* and evaluated its phylogenetic structure at the Species Genome Bin (SGB) level based on specific-species marker genes (Methods). The resulting phylogenetic tree provides interesting insights into the strain-level diversity of *L. rhamnosus* and its potential connections to probiotic use, as *L. rhamnosus* is a recognized probiotic species. We found that strains of *L. rhamnosus* were predominantly divided into two main clades, which are distinguished based on both AP status and the probiotic usage data extracted from the clinical record. We found the dominant strain primarily in infants with AP taking probiotics, and across all these samples, the *L. rhanmosus* strain seems almost identical (0.001 ~ mutation distance, Fig. 5A). Surprisingly, we also found *L. rhanmosus* strains in infants without reported probiotic use, but these strains appeared to exhibit greater diversity. One potential explanation for the probiotic strain in infants who did not use probiotics is a high prevalence of this strain in the population, and thus transmitted without probiotic use, and may accumulate mutations in this process.

Another dominant species in our analysis is *Escherichia coli*, which was the most differential between infants with AP and those without. We constructed a phylogenetic tree for *E. coli* with 120 samples from 72 infants at various ages (one month to one year) that all had sufficient coverage for *E. coli* ‘s marker genes (Fig. 5B). Although some distinct clades appeared to be enriched in either cases or controls, we found no clear overall separation between infants with AP and controls beyond a small number of samples (nodes, n < 6), making it difficult to dig deeper into the strain differences using this approach.

While strainPhlan captures strain differences based on the marker genes, we can use the deep metagenomic sequencing to explore strains beyond only the marker genes of each species. We used metagenomic assembly tools to construct metagenome-assembled genomes (MAGs; Methods, **Supp**. Figure 2), allowing us to compare the gene content differences across various clades of the same species. We generated 9,033 MAGs from 728 samples (out of the full set of 740 samples, 98.4%), which were divided into three quality categories: 3,309 high-quality MAGs (36.6%), 1,880 medium-quality MAGs (20.8%), and 3,844 low-quality MAGs (42.6%; Methods).

Next, we examined whether different MAGs were found in infants with AP vs. unaffected controls. For each species, we clustered the MAGs of that species based on similarity (using MASH distance; Methods). As expected, the MASH distance of related MAGs is statistically significantly lower compared to the unrelated MAGS (p-values < 0.05; **Supp. Table 5**), across the top 20 species with most MAGs classified to them. Overall, there was no difference between AP and controls. For some specific species, we found a statistically significant difference according to disease status. Of specific interest were the cases of *E. coli*, which was differential in infants with AP from our previous analyses[38] and showed differential MAGs here (p-value = 0.054, Fig. 5C), and *Ruminococcus B gnavus*, which has been previously associated with other allergic manifestations in infancy[27, 40], and also exhibited differential MAGs in our analysis here (p-value = 0.015, Fig. 5D; full results across all 20 species in **Supp. Table 5**). These results, together with marker-gene results demonstrating AP-associated strains raise the question of whether certain strains could serve as distinguishing markers for infants with AP and could play a role in pathogenesis.

### AP-associated *E. coli* strains are enriched for genes related to adhesion

The differences in MAGs from infants with AP compared to unaffected controls, suggests a functional difference between the two groups of MAGs, that can be further characterized by the individual genes found in each MAG. This approach will allow us to characterize genomic functional elements across strains, offering deeper insights into the distinct traits of the MAGs present in the AP or control samples. To perform a gene-level analysis we constructed pangenomes for each species of interest, enabling us to profile the presence/absence of each gene in each MAG (Methods). We next searched for sets of genes that are shared among similar MAGs that are enriched with either AP or unaffected control samples. With the resulting pangenomes, we examined shared gene clusters through phylogenetic trees produced by Roary and heatmap visualizations of gene presence-absence matrices.

To search clades of the phylogenetic tree that are dominated by infants with AP, we examined each subtree, and evaluated the AP/control distributions of its samples (found in the leaves of this subtree), comparing to the overall even distribution (50% AP) in this case-control selection of samples, using the Chi-Square test. Additionally, we evaluated whether clades in the tree were infant-oriented – consisting exclusively of samples from the same infant – or more widely distributed across individuals. In the pangenome-based phylogenetic tree of *E. coli*, we found several AP-associated clades (Fig. 5F, **Supp. Table 6**), with distinct gene groups that were shared among samples from infants with AP. This finding highlights the possibility that specific genomic features may contribute to or be influenced by AP status, underscoring the need for further research into their functional significance. To better understand the functionality of these strains, we used the EcoCyc[41] database tool, to search for enrichment in *E. coli* genes, pathways, and additional gene ontology terms. Of the 223 genes that were unique to the *E. coli* clade defined above, 150 were found in the EcoCyc database, and used for the enrichment tests (**Supp. Table 7**). One function that came up consistently in multiple enrichment tests was cell adhesion and biofilm formation (Fig. 5G). Biofilm formation and cell adhesion in commensal *E. coli* are known to have a role in healthy gut barrier formulation, enabling colonization from other beneficial anaerobes, and may influence immune development[42].

### Prediction of AP status using microbial composition

**Figure 6:**
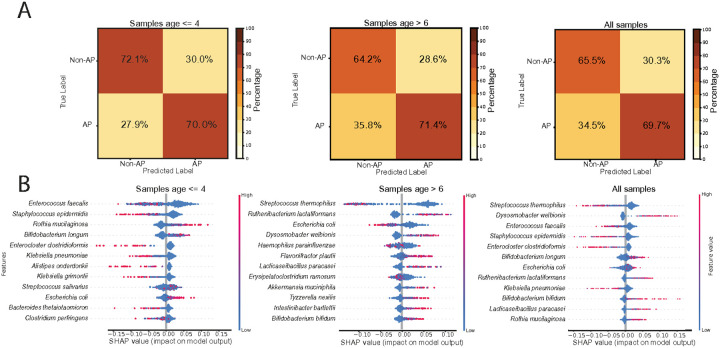
Machine learning-based classification of samples from infants with and without AP, and identification of key species-level microbial predictors **(A, B)** Random Forest classification of infants with AP and infants without AP samples using species-level features. (**A**)Confusion matrices showing classification performance stratified by visit age (≤4 months, >6 months) and for all samples combined. (**B**) SHAP summary plots illustrating the most influential species-level features contributing to model predictions in each age group and the full dataset. Each point represents an individual sample, with color indicating feature value. Feature rankings reflect the relative importance of species in distinguishing between FPIAP and control samples.

We next examined whether we can predict disease status based on the microbiome profiles. We evaluated the ability to distinguish infants with and without AP based on genus-to-species-level microbial profiles, using a Random Forest classifier (Methods). Among samples collected at age ≤ 4 months, the model correctly classified 72.1% of control samples and 70.0% of infants with AP (Fig. 6A, **left**). For samples collected after 6 months of age, accuracy was 64.2% for controls and 71.4% for infants with AP (Fig. 6A, **center**). Across all samples, overall classification rates were 65.5% for controls and 69.7% for infants with AP (Fig. 6A, **right, Supp. Table 8**).

To identify the microbial features contributing to classification, we examined SHAP values (Fig. 6B). In younger infants (Fig. 6B, **left**), control-associated species included *Enterococcus faecalis, Staphylococcus epidermidis, Klebsiella pneumoniae*, and *Clostridium perfringens*, while AP-associated species included *Rothia mucilaginosa, Bifidobacterium longum, Streptococcus salivarius*, and *Escherichia coli*. In older infants (Fig. 6B, **cente**r), control-associated species included *Streptococcus thermophilus* and *Escherichia coli*, while AP-associated species included *Ruthenibacterium lactatiformans, Dysosmobacter welbionis, Flavonifractor plautii*, and *Bifidobacterium bifidum*. When considering all samples (Fig. 6B, **right**), control-associated species included *Streptococcus thermophilus, Enterococcus faecalis, Staphylococcus epidermidis*, and *Klebsiella pneumoniae*, while AP-associated species included *Dysosmobacter welbionis, Bifidobacterium longum, Escherichia coli*, and *Bifidobacterium bifidum*.

At the genus level, SHAP values revealed that in infants aged ≤ 4 months, *Klebsiella, Enterococcus*, and *Staphylococcus* were the top contributors to classification, with *Bifidobacterium, Blautia*, *Parabacteroides*, and *Streptococcus* also playing important roles. In older infants (> 6 months), classification was primarily driven by *Streptococcus, Enterocloster, Ruthenibacterium*, and *Dysosmobacter*, along with contributions from *Escherichia, Actinomyces*, and *Intestinibacter*. In the full dataset, top-ranking genera included *Enterocloster, Klebsiella, Dysosmobacter, Enterococcus, Bifidobacterium*, and *Blautia*, as well as *Veillonella, Actinomyces*, and *Staphylococcus* (**Supp. Table 9**).

These results were obtained after filtering out *Lacticaseibacillus rhamnosus*, a probiotic species commonly administered to infants with AP. In the unfiltered dataset, *L. rhamnosus* had one of the highest SHAP values among AP-associated taxa, strongly influencing classification. However, its abundance likely reflects therapeutic exposure rather than underlying microbial differences. To avoid confounding, this feature was excluded from the final analyses (**Supp.** Figure 4, **Supp. Table 10,11**).

## Conclusions

In this study, we used longitudinal metagenomic sequencing of 740 stool samples from 163 infants to investigate the gut microbiome in food protein-induced allergic proctocolitis (AP). To our knowledge, this is the largest longitudinal metagenomic analysis of infants with AP. By integrating high-resolution species- and strain-level data with detailed clinical metadata, we examined microbial dynamics across infants’ first year of life, including before, during, and after symptom onset and resolution compared to carefully age matched unaffected controls from the GMAP study.

Infants with AP exhibited less stable microbiomes during the early pre-symptomatic transition from 2 weeks to 1 month of age. Species-level modeling revealed that *Escherichia coli* and *Bifidobacterium bifidum* were significantly enriched in infants with AP during the first two months of life, while *Clostridium paraputrificum*, *Klebsiella michiganensis*, and *Bacteroides* were more abundant in infants without AP across multiple stages. Additionally, *Bifidobacterium dentium* and *Tyzzerella nexilis* were enriched in AP during the resolved stage.

Functional pathway analyses revealed an enrichment of the O-antigen biosynthesis pathway of *E. coli* in infants with AP during the symptomatic stage, particularly interesting given the enrichment of E.coli in AP and worth further study given other evidence for the role of LPS in food allergy[43]. Pyruvate fermentation to propionate was consistently reduced in AP infants during early, pre-symptomatic, and symptomatic stages but then significantly enriched following symptom resolution, a particularly compelling pattern as it correlates with clinical status. After symptom resolution, succinate fermentation to butyrate and acetyl-CoA fermentation to butyrate pathways were significantly more abundant in infants with AP, consistent with altered SCFA metabolism. In contrast, phytol degradation and glycogen degradation I pathways were reduced in infants with AP across multiple stages. These findings highlight disease-specific shifts in microbial metabolism and functional activity during and after AP.

Strain-level analyses uncovered additional disease-linked patterns. *E. coli* showed different strains clades based on both marker gene phylogenies and mash distance between MAGs (metagenome-assembled genomes) indicating the existence of AP-enriched lineages. Notably, *E. coli* MAGs from infants with AP exhibited greater similarity to one another compared to those from unaffected control infants. Functional analysis of the AP-associated *E. coli* MAGs revealed significant enrichment of genes involved in biofilm formation and cell adhesion processes. This finding is particularly relevant given the established connection between cell adhesion, inflammatory responses, and immune-mediated pathways in disease pathogenesis. *Lacticaseibacillus rhamnosus* strains were strongly associated with probiotic use and predominantly found in infants with AP, suggesting probiotic exposure does influence microbial strain composition.

Machine learning further supported the presence of a disease-associated microbial signal. A Random Forest classifier trained on species-level features distinguished infants with and without AP with moderate accuracy, particularly at younger ages. Key AP-associated taxa included *L. rhamnosus, B. longum, B. bifidum*, and *E. coli*, while samples of infants without AP were more frequently associated with Klebsiella pneumoniae, B. breve, and various Clostridium species.

Together, our findings reveal that infants with AP harbor distinct microbial compositions and strain-level lineages, particularly in early life and around disease onset, suggesting a potentially important role for the microbiome and microbial functions in disease pathogenesis. This work lays a foundation for future efforts to further classify early microbial biomarkers and novel interventional targets for AP, which also represents an understudied opportunity for life-threatening IgE-mediated food allergy prevention.

## Methods

### Cohort Selection and Study Design

This study was conducted within the Gastrointestinal Microbiome and Allergic Proctocolitis (GMAP) cohort, a prospective observational study designed to investigate the development of the early-life gut microbiome and its association with pediatric allergic diseases. The GMAP study was approved by the Massachusetts General Hospital Institutional Review Board (2013P002374), and a parent of all enrolled infants gave written informed consent.

From this larger cohort, we identified 84 infants with a clinical diagnosis of AP and 79 age-matched infants without AP. All 163 infants were matched by age and sampling density, and each contributed a median of four longitudinal stool samples during the first year of life. Diagnosis of AP was confirmed via standardized chart review and based on the presence of gross or guaiac-positive bloody stools, consistent with previously published criteria[17].

In total, 740 stool samples were subjected to deep, paired-end metagenomic sequencing (median ~14 million paired reads per sample). Based on clinical documentation, three distinct disease states were defined for each infant with AP: Pre-symptomatic (prior to symptom onset), Symptomatic (during symptomatic period), and Resolved (post-resolution), enabling stratified assessment of microbial changes across the disease course. Demographic and clinical metadata, including delivery mode, feeding type, sex, and probiotic use, were systematically collected.

### Sample Collection and Processing

Fresh stool samples were collected from infant diapers during routine pediatric visits using standardized procedures. Study staff transferred stool into sterile collection tubes on-site using sterile spatulas, following established protocols. Samples were immediately stored at −20°C at the clinic and later transferred to −80°C for long-term storage until extraction.

DNA was extracted from approximately 100–150 mg of frozen stool using the Qiagen DNeasy PowerSoil HTP 96 Kit (Catalog no. 12955–4), following the manufacturer’s protocol. Samples were bead-beaten on a TissueLyser II (Qiagen) at 20 Hz for 10 minutes to ensure lysis of diverse microbial cell types. Following extraction, DNA was quantified using Qubit fluorometric quantification (Thermo Fisher Scientific) and normalized prior to library preparation.

### Whole-Metagenome Shotgun Sequencing and Profiling

Metagenomic libraries were constructed using the Illumina DNA Prep Kit and sequenced on an Illumina NovaSeq 6000 platform to generate paired-end 150 bp reads. Sequencing was performed to a median depth of ~14 million paired-end reads per sample. Adapter sequences and low-quality reads were removed using fastq, and host (human) reads were filtered by alignment to the GRCh38 reference genome using Bowtie2[44].

Microbial taxonomic profiling was performed with MetaPhlAn4[45], which uses clade-specific marker genes to provide species- and strain-level resolution across metagenomic samples.

### Microbiome Composition Analyses

To approximate standard pediatric visit intervals (typically scheduled at birth, 0.5, 1, 2, 4, 6, 9, and 12 months), we defined corresponding age bins centered around these target time points. To account for natural variation in sampling times around scheduled visits, bin edges were refined based on the empirical age distribution, ensuring that samples collected slightly before or after a target time point were appropriately grouped. The final bin thresholds used were: 0, 0.35, 0.75, 1.5, 3, 5, 8, 10.5, and 14 months.

### Taxonomic Profile Analysis

We identified the 15 most abundant taxa across all samples, classifying remaining taxa collectively as “Others.” Relative abundance profiles were visualized using stacked bar plots stratified by clinical group (infants with AP versus infants without AP) and age bins. Profiles were generated separately at the genus and species levels.

### Principal Coordinates Analysis (PCoA)

Global microbiome composition was visualized through principal coordinates analysis (PCoA) using Aitchison distances. Sample points were color-coded by symptom group, age bin, Chao1 richness, and Shannon diversity. ANOVA was used to test group-level separation along the first principal coordinate axis. Bray–Curtis-based PCoA was also performed using the top 15 genera to explore specific shifts in relative abundance (**Supplementary Fig. 3A-B**).

### Alpha Diversity

Alpha diversity was quantified using the Chao1 index (richness) and Shannon index (richness and evenness). To assess diversity trends over time, Pearson correlation coefficients were calculated between each diversity index and infant age (as a continuous variable). Samples were also stratified by clinical stage (Pre-symptomatic, Symptomatic, Resolved, Control) within each age bin, and diversity distributions were visualized using boxplots. Pairwise t-tests were used to assess statistical significance among symptom groups.

### Beta Diversity and Microbiome Stability

Microbial community stability was evaluated by calculating Bray–Curtis distances. Two analyses were performed:

1. Stability was compared within infants (longitudinal sample pairs) and between infants (samples from different infants) across age transitions.
2. Within-infant stability was compared between infants with AP and unaffected controls using t-tests at key age transitions.

### Definition of Disease-State Sample Subsets

To examine microbial differences related to disease progression, we defined three subsets for each infant with AP:

1. Last Pre-symptomatic sample — the final sample prior to symptom onset
2. First Symptomatic sample — the first sample collected during symptomatic period
3. First Resolved sample — the first sample collected after clinical symptom resolution

To reduce sampling bias, we included only one sample per infant per disease phase. Each selected sample from infants with AP was matched to a sample from an infant without AP by infant age.

Matching was performed using the Hungarian method (linear sum assignment). A cost matrix of absolute age differences was constructed, and one-to-one matches were enforced using the solve_LSAP function in R’s “clue” package (v0.3–63), ensuring tight age alignment for downstream MaAsLin2 analysis. (**Supplementary Fig. 3C**)

### Differential Abundance Analysis

Differentially abundant taxa were identified using multivariable linear modeling via MaAsLin2. Fixed effects included symptom group, mode of delivery, age at visit, and probiotic use. Abundances were arcsine square-root transformed prior to modeling. Taxa were filtered to retain only those with ≥0.1% relative abundance. Statistical significance was set at p < 0.05, and results were adjusted for multiple testing using the Benjamini–Hochberg procedure (FDR); taxa with q < 0.25 were considered significant.

### Functional Pathway Profiling

To investigate microbial functional differences between infants with and without AP, we used HUMAnN 3.0 to process metagenomic FASTQ files, leveraging MetaPhlAn 4-generated taxonomic profiles to improve mapping accuracy. Pathway abundances were quantified using the UniRef90 database and normalized with HUMAnN’s renorm utility. Bacteria-stratified abundances were collapsed to obtain total pathway-level values.

Statistical analyses were performed using MaAsLin2, with covariates matched to those used in the taxonomic models. Analyses were stratified by sample subsets, including the full cohort, early-life samples (≤2 months), and age-matched clinical stages (pre-symptomatic, symptomatic, resolved). Significance was defined as p < 0.05 and q < 0.25.

### Machine Learning

We trained a Random Forest classifier to distinguish infants with AP from infants without AP using species- and genus-level taxonomic features. Features were filtered to include only those with a mean relative abundance >1%, and all retained features were standardized. Class imbalance was corrected via weighted training.

To avoid confounding due to therapeutic exposure, we specifically filtered out *Lacticaseibacillus rhamnosus* - a probiotic species commonly used in the treatment of AP. Since this bacterium is often administered to symptomatic infants as part of their therapy, its presence is expected to be higher in AP samples and may not reflect endogenous microbiome patterns. Including it could artificially inflate model performance or distort SHAP-based interpretation of feature importance.

Feature selection was performed iteratively by removing the least informative features (based on Gini importance), retaining the top 120. Model evaluation used 5-fold cross-validation. Optimized hyperparameters were tuned separately for three groups: all samples, ≤4-month samples, and >6-month samples.

SHAP (SHapley Additive exPlanations) values were computed with TreeExplainer to assess feature importance. All modeling was implemented in Python 3.9 using pandas, scikit-learn, NumPy, and shap.

### Strain level analysis

Strain level analysis was performed using StrainPhlan[46] with default parameters, and phylophlan_mode set to be “accurate”. Metadata used to annotate the tree based on different traits. Probiotic consumption feature was generated by integrating all types of probiotic consumption information from questionnaires and metadata.

### Metagenomic Assembled Genomes Generation

Metagenomic assembled genomes (MAGs) were created by the following pipeline: Genome assembly, binning, quality assessment, taxonomic classification of assemblies and coverage estimation. Genome binning was performed using Metaspades [47]. The resulting contigs were then mapped to their original reads with minimap2 [48]. MetaBat2 [49] which uses sequence composition and coverage information, was used to bin probable genomes using default parameters. CheckM2 [50] was used on all binned genomes to confirm completeness and contamination scores. Chosen threshold for high quality of bins were genomes with a quality of completeness >= 85 and contamination < 5. Bins with 50 <= completeness < 85 and contamination < 5 were considered medium quality. Genome bins were analysed for their closest taxonomic annotation. To perform Taxonomic assignment, the genome set was assigned into the microbial tree of life using GTDB [51] to identify the closest ancestor and obtain a putative taxonomy assignment for each genome bin. The Relative abundance of MAGs was calculated using coverM [52]. Later on a comparison of correlation between relative abundance of MAGs and relative abundance of samples was done to reassure taxonomic classification.

### Mash Distance Calculation

Mash[53] distance was calculated using Mash tool. Parameters: mash sketch -o “$MASH_PATH/sketches_folder/${base_name%.fa}” “$file”. to search for groups of similar MAGs within any given species, we calculated the Mash distances across all MAGs which were classified to this species. This calculation was conducted across the top 20 species with most MAGs classified to them. We then analyzed the distribution of these Mash distances of unrelated samples (of different infants) and related samples (of the same infant), and further within the related samples, differentiating between infants with and without AP.

### Pangenome Construction

With the generated MAGs, a Pangenome was created for selected species using Prokka [54] tool to annotate the MAGs and Roary[55] tool to create the Pangenome with default parameters. Roary also generated the Phylogenetic tree of the MAGs annotation.

### Gene enrichment analysis

Relevant genes were analyzed using BioCyc databases[56–58]. Smart tables of gene enrichments were created based on gene list files from Roary results. Genes were chosen based on the Roray results of clusters in the pangenome, when genes from same samples clusters of infants with/without AP were analyzed together for enrichment patterns.

## Supplementary Material

Supplementary Files

This is a list of supplementary files associated with this preprint. Click to download.

- SuppTable1StabilitymeasurementsofmicrobiomeinrelatedsamplesvsunrelatedsamplesandAPvs.unaffectedcontrolsofthesameinfantsamples.xlsx
- SuppTable2DifferentialtaxabetweeninfantswithAPandunaffectedcontrolsgenuslevel.xlsx
- SuppTable3DifferentialtaxabetweeninfantswithAPandunaffectedcontrolsspecieslevel.xlsx
- SuppTable4DifferentialpathwaysbetweeninfantswithAPandunaffectedcontrols.xlsx
- SuppTable5MashdistancestatisticsacrossMAGsofthesamespecies.xlsx
- SuppTable6RoaryE.colisubtreesinternalAPunaffectedcontroldistributionofsamples.xlsx
- SuppTable7EnrichmentofbiologicalprocessesinE.coliMAGsfoundtobeenrichedininfantswithAP.xlsx
- SuppTable8Performanceevaluationofrandomforestpredictionmodels.xlsx
- SuppTable9SHAPresultsforAPpredictionmodelsongenusandspecieslevelinputs.xlsx
- SuppTable10PerformanceevaluationofrandomforestpredictionmodelswithLacticaseibacillusrhamnosus.xlsx
- SuppTable11SHAPresultsforAPpredictionmodelsongenusandspecieslevelinputswithLacticaseibacillusrhamnosus.xlsx
- SupplementaryFigure1Extendedoverviewofmicrobiomecompositiondiversitystructureandsamplematchingacrossthefirstyearoflife..pdf
- SupplementaryFigure2OverviewofMAGscreationprocessandquality.pdf
- SupplementaryFigure3.MicrobiomestructureandagematchingstrategyforMaAsLin2analysis..pdf
- SupplementaryFigure4MachinelearningbasedclassificationofsamplesfrominfantswithandwithoutAPandidentificationofkeyspecieslevelmicrobialpredictor
- SupplementaryLegends.docx
